# Vulvar Crohn's disease as extra intestinal manifestation

**DOI:** 10.11604/pamj.2020.37.366.26028

**Published:** 2020-12-22

**Authors:** Fatima El Mangoub, Jaouad Kouach

**Affiliations:** 1 Department of Gynecology-Obstetric, Military Training Hospital Mohamed V, University of Medicine, Rabat, Morocco

**Keywords:** Vulvar Crohn’s disease, vulvar ulceration, vulvar hypertrophic, extraintestinal manifestation of Crohn’s disease

## Image in medicine

Crohn's disease is a chronic granulomatous inflammatory disorder that usually affects the gastrointestinal tract. It may be associated with extra-intestinal lesions such as genital female manifestations. The most common clinical manifestation of vulvar Crohn's disease is: vulvar swelling, edema; affecting both labia minora and majora or the vaginal wall; ulceration; hypertrophic lesions; chronic suppuration; vulvar abscesses. Diagnosis of vulvar Crohn's disease is confirmed on histopathology by the presence of inflammatory infiltrates, epidermal ulceration, and non caseating granulomas. Its progress is unpredictable; while some lesions resolve spontaneously, others fail both medical management and surgical debridement. There are no well-established treatment strategies of vulvar Crohn's disease. It consists on a prolonged course of antibiotherapy (classically metronidazole), often in combination with immunosuppressive medications including topical steroids and monoclonal antibody therapy (Tumor necrosis factor (TNF) inhibitors). We report a case of a 45 years old women, three gravida, and three para. In her medical history, we found Crohn's disease since fifteen years. She was treated by antibiotics (metronidazole) and topical steroids medications. She was referred to our department for management of vulvar lesions (A, B, C) without any gastrointestinal manifestations. On physical examination she was hemodynamically stable, she had painful vulvovaginal hypertrophic lesions (A, B), vulvar ulcers “knife-cut” ulcers (C). Vulvar Crohn's disease was suspected and biopsies of genital lesions were performed to confirm the diagnosis. The histopathological examination showed the presence of inflammatory infiltrates, epidermal ulceration, and non-necrotizing granulomas. Her gastroenterologist was contacted and the decision was to put her on immunosuppressants (azathioprine) in combination with topical steroids treatment. She was seen again at 3 and 9 months of treatment with partial regression of the vulvovaginal lesions. Surgical treatment was offered due to the severity of the lesions and the partial effectiveness of the medical treatment, but it was refused by the patient. Diagnosis of vulvar Crohn's disease may be difficult especially when this manifestation is the first symptom. In this case, vulvar Crohn's disease must be differentiated to non-infectious causes of genital ulcers and to infectious causes include cutaneous abscesses. The diagnosis is confirmed on histopathology. Treatment options are not yet well codified.

**Figure 1:**
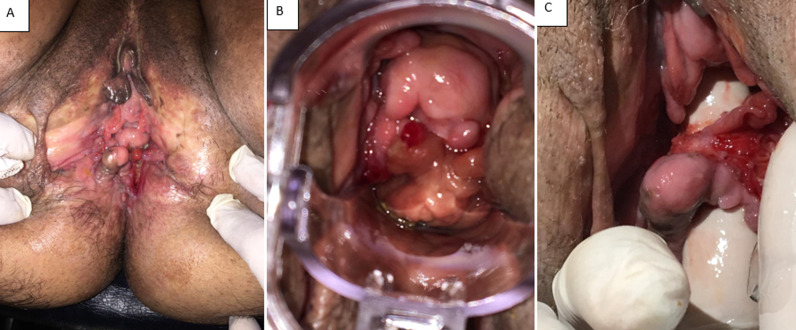
vulvovaginal Crohn's disease as extra intestinal manifestation: A) vulvar hypertrophic lesions affected labia minora; B) hypertrophic lesions affected vaginal wall; C) vulvovaginal ulcers

